# Characteristics of Patient Safety Incident Occurrences Reported by Japanese Homecare Nurses: A Prospective Observational Study

**DOI:** 10.3390/nursrep11040090

**Published:** 2021-12-14

**Authors:** Natsuki Yamamoto-Takiguchi, Takashi Naruse, Mahiro Fujisaki-Sueda-Sakai, Noriko Yamamoto-Mitani

**Affiliations:** 1 Faculty of Nursing and Medical Care, Keio University, Kanagawa 252-0883, Japan; 2 Global Nursing Research Center, Graduate School of Medicine, The University of Tokyo, Tokyo 113-0033, Japan; takanaruse@g.ecc.u-tokyo.ac.jp; 3 Department of Public Health Nursing, Division of Health Sciences, Graduate School of Medicine, Tohoku University, Sendai 980-8575, Japan; mahiro.fujisaki@med.tohoku.ac.jp; 4 Department of Gerontological Home Care and Long-Term Care Nursing/Palliative Care Nursing, Division of Health Sciences & Nursing, Graduate School of Medicine, The University of Tokyo, Tokyo 113-0033, Japan; noriko-tky@g.ecc.u-tokyo.ac.jp

**Keywords:** homecare, homecare nursing, occurrence, patient safety, patient safety incident, prospective observational study

## Abstract

Patient safety incidents (PSIs) prevention is important in healthcare because PSIs affect patients negatively and increase medical costs and resource use. However, PSI knowledge in homecare is limited. To analyze patient safety issues and strategies, we aimed to identify the characteristics and contexts of PSI occurrences in homecare settings. A prospective observational study was conducted between July and November 2017 at 27 Japanese homecare nurse (HCN) agencies. HCNs at each agency voluntarily completed PSI reports indicating whether they contributed to PSIs or were informed of a PSI by the client/informal caregiver/other care provider during a period of three months. A total of 139 PSIs were analyzed, with the most common being falls (43.9%), followed by medication errors (25.2%). Among the PSIs reported to the HCN agencies, 44 were recorded on formal incident report forms, whereas 95 were reported as PSIs that required a response (e.g., injury care) but were not recorded on formal incident report forms. Most PSIs that occurred when no HCN was visiting were not recorded as incident reports (82.1%). Developing a framework/system that can accumulate, analyze, and share information on PSIs that occur in the absence of HCNs may provide insights into PSIs experienced by HCN clients.

## 1. Introduction

The aging and deinstitutionalization of the Japanese population have led to a rapid increase in the demand for homecare services [1]; homecare nursing (HCN) services, with approximately 580,000 clients in Japan [2], play an important role in homecare. In Japan, potential clients can apply with an HCN agency through their primary care physician or directly, and if their physician recognizes that an HCN service is required, they will issue an “Order for visiting nursing.” HCN services will then be provided based on the creation of a care plan [3]. The main diseases/injuries among HCN users are cerebrovascular diseases (15.4%), musculoskeletal diseases (9.0%), dementia (including Alzheimer’s disease) (8.6%), malignant neoplasm (8.3%) [4]. As service demands increase rapidly, the maintenance and improvement of the quality of these services have become important issues in Japanese homecare.

Patient safety is an essential factor contributing to the quality of healthcare services [5], and patient safety has been well studied in acute care hospitals [6]. Patient safety incidents (PSIs) are defined as events or circumstances that could result, or did result, in unnecessary harm to a patient [7]. Since PSIs can affect both the activities of daily living (ADL) and the quality of life of patients (clients) and may lead to an increase in medical costs and resource use [8,9], preventing PSIs in healthcare settings is essential.

PSIs are not only reported and accumulated, but also analyzed, and lessons learned are disseminated within an organization and to other organizations with similar risks, thereby contributing to improved patient safety in health care services [10]. PSI reporting is the first step that can lead to (1) early warnings about new serious risks, (2) dissemination of knowledge gathered from investigating serious events, (3) clarification of potential risks through quantitative analysis of reported data, and (4) insights into underlying system deficiencies [11]. However, studies to improve patient safety, such as those exploring PSIs occurring frequently, are primarily conducted in medical institution settings [6], and despite the growing need for homecare, there is insufficient understanding of the characteristics of patient safety in homecare settings.

In addition, a previous study of a chart review of PSIs in homecare services indicated that the percentage of documented incident reports was as low as 14.8% of adverse events recorded in client charts [12]. The lack of documentation of PSIs that occur as incident reports may be an obstacle to the quantitative collection and analysis of PSIs, which may lead to the underestimation of PSIs and inhibit the consideration of effective preventive measures against PSIs.

Reasons for PSIs not being recorded as incident reports have been pointed out in medical institution settings, such as the lack of definition of events to be recorded in the organization, inappropriate content of the report form, or fear of being reprimanded [13].

However, a qualitative study [14] investigating homecare nurses’ perceptions of and attitudes toward reporting PSIs suggests that the context in which a PSI occurs might determine whether the HCN services’ PSI is recorded as an incident report. If a client falls and sustains fractures while standing up on their own during an HCN visit, an incident report will be filed, as it is carried out at the hospital. Conversely, if the same incident occurs when the HCN is not visiting the client at home, it is not recorded as an incident report and information is shared as part of the client’s condition change in the HCN agency. Therefore, a previous study [15] that only collected and analyzed PSIs recorded as incident reports in homecare nursing settings may have underestimated the actual PSIs experienced by HCN clients. Previous studies have used chart reviews [12,16] or self-reported PSIs in the past year [15], however, identifying unrecorded events is difficult when using chart reviews, and retrospective studies are limited by recall bias.

In our previous study [14], we qualitatively clarified the definition of PSI that HCNs recognize and report to the HCN agencies, and whether or not it is recorded as an incident. However, it is not clear quantitatively how many PSIs actually occur among clients of HCN services and what kind of PSIs are recorded as incidents. To discuss PSI prevention strategies in homecare settings, this study aimed to collect PSIs reported in HCN agencies to understand (1) what kinds of PSIs occur and (2) whether PSIs are recorded as incident reports in homecare settings.

## 2. Materials and Methods

### 2.1. Definitions

In this study, a PSI was defined as an event that could have resulted, or did result, in unnecessary direct harm to a client’s condition (excluding deterioration of underlying conditions and complications in the client with the passage of time). This definition was based on the results of previous qualitative research in which we interviewed 16 Japanese HCNs [14].

### 2.2. Design and Procedure

This prospective observational study is reported according to the STROBE statement [17].

The present study was conducted in HCN agencies in a city (with a population greater than 500,000 people) in the Kyushu region of Japan. Anonymous questionnaires were completed by nurses in selected HCN agencies between July and November 2017. We mailed documents describing the study design to all 84 HCN agencies within the city. If a manager of an agency agreed to participate, the first author visited their agency and explained the study methods to all staff directly using a study description document. The managers and HCNs were asked to complete self-administered anonymous questionnaires about the agency or on personal information; report sheets on PSIs (five sheets) were also given to all HCNs (including managers) in the participating agencies. The PSI report sheet consisted of a self-administered anonymous form for recording information related to PSIs that occurred; each PSI was recorded on a separate sheet. When a manager/homecare nurse observed a PSI during the study period (three months after the first author visited the agency), they were required to report it using this sheet. Completed PSI report sheets were mailed directly to the researchers every 30 days. The questionnaire was distributed only to those who provided informed consent, and participants could contact the researchers at any time to withdraw their consent to participate.

### 2.3. Measurements

#### 2.3.1. HCN Agency Variables

Agency variables included the number of full-time equivalent HCN staff and the total number of PSIs reported in the previous year. The summed number of PSIs reported during the study period (three months) was calculated for each agency by the first author.

#### 2.3.2. HCN Variables

HCN variables included age, sex, position in the agency (manager, chief, or staff), length of service as an HCN, the average number of visits per day, number of incident reports completed over the previous year, recognition of the effectiveness of sharing PSI information between HCN agencies for patient safety (effective/not effective), and experience sharing PSI information with other HCN agencies (yes/no).

#### 2.3.3. PSI Variables

PSI variables included the types of PSIs (i.e., falls, injury without fall, occurrence or deterioration of pressure ulcer, medication error, etc.), the context of the PSI occurrence (whether the HCN was at the client’s home when the PSI occurred; i.e., presence or absence of HCN), the impact of the PSI on the client (whether unplanned admission/consultation was required due to its occurrence), the individual (HCN, client, informal caregiver, or other care professional) who mainly contributed to the occurrence of the PSI, the person who identified and informed the HCN of the PSI occurrence (HCN, client, informal caregiver, or other care professional), reporting form of PSIs (“formal” = occurrence reported and recorded as an incident report form or “informal” = occurrence reported in a staff meeting or recorded in a client’s medical chart but not recorded as an incident report form), and the demographic variables of clients who experienced PSIs (age, sex, and ADL). In terms of ADL, HCNs answered whether clients who experienced PSI were able to move about and defecate independently or whether they needed assistance (independence or need someone to watch over/assist). Whether the same patient experienced multiple PSIs during the study period was noted in the Summary of Incidents Occurred column of the PSI report sheet.

### 2.4. Statistical Analysis

Descriptive statistics were used to detail the characteristics of HCN agencies, HCNs, and clients who experienced PSIs. Continuous variables were characterized using means and standard deviations (SD), whereas categorical variables were characterized using the number (n) and percentages. A previous qualitative study [14] suggested that PSIs collected with the same definition might include some that are recorded as incidents and some that are not. Therefore, to examine the difference between a PSI that is recorded as an incident report and one that is not, unpaired t-, χ^2^, and Fisher’s exact tests were performed to compare the PSI variables according to the reporting form of PSIs (formal vs. informal) reported to HCN agencies. The criterion for statistical significance was set at *p* < 0.05. IBM-SPSS ver. 26.0 was used for all statistical analyses.

## 3. Results

**Figure 1:**
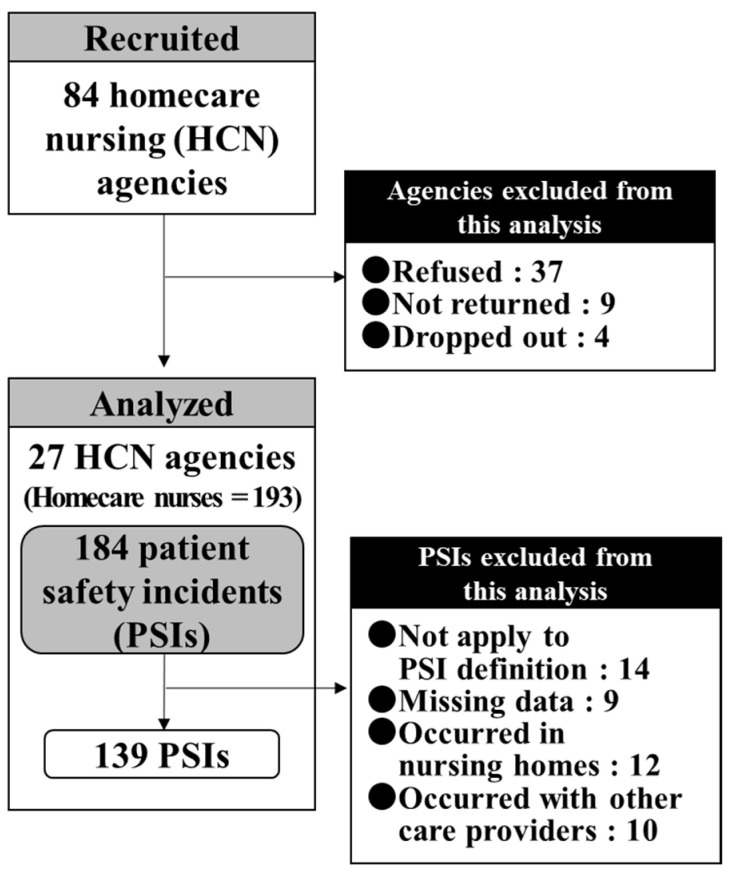
Flow chart of study recruitment and participation.

The study recruitment and participation processes are illustrated in Figure 1. Of the 84 HCN agencies contacted for this study, 37 refused to participate, 16 did not respond, and four dropped out during the three-month study period. Of the 184 PSIs reported during the study period, 14 were not applicable to this study’s definition of PSI (e.g., deterioration of an underlying condition of the client with the passage of time), and nine PSIs with over 50% missing data were excluded from the analysis, 12 PSIs occurred in nursing homes, and 10 PSIs occurred with other care providers as contributors. Therefore, the final analysis was conducted using data on 139 PSIs from 27 HCN agencies (HCNs = 193) during the three-month study period (total response rate of agencies: 32.1%; total valid response rate of PSI report sheet: 75.5%).

Table 1 shows the characteristics of the 27 HCN agencies and the 193 HCNs. The HCN agencies had an average of 6.3 full-time equivalent staff members (SD: 2.9; range: 2.5–12.0), an average of 3.8 visits per day per staff member (SD: 2.0; range: 0.5–20.0), and an average of 6.3 PSIs reported (SD: 5.8; range: 0–26.0). The average age of the HCNs was 45.5 years (SD: 8.0 years; range: 26–69 years), the average length of service as an HCN was 7.9 years (SD: 6.1 years; range: 1–23 years), and the average number of HCN visits per day was 3.8 (SD: 2.0; range: 0.5–20). Most of the participants reported that they had completed 0–5 (86.0%) incident reports in the previous year, whereas 94.5% of the HCNs recognized that sharing PSI information among HCN agencies facilitates patient safety, with 28.5% of the participants having actually shared PSI information with other HCN agencies.

Table 2 shows a comparison of the form of reporting of PSIs between cases recorded as incident reports (formal) and those not recorded as incident reports (informal). Eight clients experienced two or more PSIs. The most common PSI type was falls (43.9%), one-fifth of PSIs required unplanned admission/consultation caused by their occurrence (20.9%), and clients’/informal caregivers’ decisions or actions contributed to the occurrence of 75.5% of PSIs, and the clients/informal caregivers were the most frequent PSI identifiers (56.8%). Among the PSIs reported to the HCN agencies, 44 (31.7%) were recorded on incident report forms, and of the 29 cases that required unplanned admission/consultation at hospital/clinic, 21 (72.4%) were not recorded on incident report forms. Of the 21 incidents that required unplanned admission/consultation at hospital/clinic with no documented incident reports, 16 (76.2%) were PSIs that occurred in the absence of the HCN. Meanwhile, 95 PSIs (68.3%) were reported to the HCN agencies, and necessary actions (i.e., care for injuries, clients’ medical records, and information sharing among agency staff) were taken but not recorded on the incident report forms. The majority of the PSIs that occurred in the absence of HCNs were not recorded as incident reports (82.1%), and the majority of the PSIs whose contributors were clients/informal caregivers were not recorded as incident reports (89.5%).

## 4. Discussion

The present study prospectively investigated PSI characteristics occurring among clients utilizing HCN services. The average number of full-time equivalent staff and the average number of visits per day per staff member showed similar trends as those reported in Japanese national surveys on long-term care service facilities and agencies [18,19]. The average age and average length of service as an HCN tended to be similar to those published in a large-scale survey of visiting nurses [20]. The average age of clients and sex were also similar to those in Japanese national surveys [18]. Because of the similarities, we believe that our data can show a representative trend of general HCN agencies in Japan. An average of two PSIs was reported each month by each HCN agency. During the 3-month observation period, more than half of the PSIs occurred when the HCN was not visiting a client, and these were not recorded as incident reports; PSIs, which were precipitated by the client/informal caregiver’s decisions or actions were the most common.

Similar to the findings of previous studies in developed countries [7,16], the findings of this study suggest that falls are the most common incidents among HCN agency clients. Furthermore, in this study, most of the falls among HCN agency clients occurred when HCNs were absent (91.8%). Falls are common among older people and cause tremendous morbidity, mortality, and healthcare service use [21]. The findings of the present study suggest that in homecare settings, it is possible that most of these falls occur when a care provider is not present, suggesting the importance of identifying PSIs that occur when a care provider is not at the patient’s home. Even in hospitals, it is possible for an inpatient to fall in a hospital room without a care provider present. However, the time required for the fall to be detected by the care provider and assistance to be provided is likely to be longer in a homecare environment where the client and HCN are in separate locations than in an institutional environment where the patient and care provider are in the same institution. Since delays in detecting and responding to PSIs that occur in the absence of caregivers can lead to serious illness or even death of clients, homecare providers need to not only consider effective preventive measures but also to establish a system that can detect and respond to PSIs as quickly as possible.

To prevent falls among older adults living at home, individual physical and environmental fall risk assessments, continuous functional training based on these assessments, and installation of welfare equipment, such as handrails, have been employed throughout Japan [22]. For effective risk assessment of falls and identification of preventive measures, as well as to ensure that associated parties, including clients and their families, have opportunities to consider preventive measures, establishing a system that easily allows clients/families to share complete information on falls occurring in the absence of caregivers to related professions may be important. In addition, the development of a system that can accumulate and share information on the preventive measures implemented for each fall case and evaluate their effectiveness may improve the quality of understanding and response to falls that occur in HCN settings.

Although falls occurred most frequently in the study as a whole, medication errors occurred more frequently than falls when considering PSIs recorded as incident reports. Among the medication errors recorded as incident reports, we observed cases such as not distributing the oral medication for the required number of days until the next visit or administering the wrong medication. HCNs have a set amount of time available for service provision according to the care requirements of their clients [23], and they are required to provide appropriate care within that time frame. Therefore, they may feel the complexity of the management procedures, such as when there are large numbers of medications and the time pressure to provide adequate care within the stipulated time. Since cumbersome procedures or time pressure are some of the main factors that cause unsafe behavior [24], there is a need for organizational improvements, such as simplification of medication distribution procedures and review of the visit schedules in HCN agencies.

Consistent with the findings of a previous qualitative study [14], PSIs that occurred when HCNs were not visiting were generally not recorded as incident reports. However, because clients spend more time in their lives without an HCN [25], it might be difficult to understand the PSIs they are experiencing and consider effective countermeasures if the PSI during that time is not recorded in the form of an incident report. Therefore, a framework/system needs to be developed that can collect information on PSIs experienced by HCN clients, including PSIs that occur in the absence of an HCN.

Clients’/informal caregivers’ decisions or actions (PSI case example: during the absence of an HCN, a client with a high risk of falling fell as a result of walking without assistive devices because they were too lazy to use them at home) contributed to 75.5% of the PSIs, which is consistent with a previous study indicating that clients’ and informal caregivers’ behavior contributed to 68.8% of adverse events in homecare [12]. Patients easily adapt to interventions and restrictions by care providers in hospital settings [26]; furthermore, it is easier to maintain patients’ adherence to care and medication in hospitals. Conversely, although HCNs can offer health education and recommend suggestions for care in homecare settings, it is ultimately up to the clients and informal caregivers to implement these recommendations [6]. Therefore, a client’s adherence to care and medication at home might differ from that in hospital settings, which can lead to PSIs such as untreated conditions or forgetting to take medication. Our findings suggest that when trying to address patient safety in homecare, HCNs face concerns in terms of not only the involvement of clients and informal caregivers but also balancing between interventions for safety and personal autonomy.

## 5. Conclusions

The most common PSIs experienced by clients of HCN services were falls, followed by medication errors. Despite collecting PSIs using the same definition, PSIs included those that were recorded on formal incident report forms and those that were not. Many PSIs occurred in the absence of HCNs and were not recorded as incidents, while they were reported to the HCN agency and necessary actions (i.e., care for injuries) were taken. The amount of time when HCNs are not present at a client’s home is longer than that of their presence at the client’s home. Therefore, the development of a framework/system that can also accumulate, analyze, and share information on PSIs that occur when HCNs are not present at the client’s home may lead to a better understanding of the PSIs experienced by clients and the creation of effective preventive measures for homecare settings. For example, client/caregiver and care providers can establish protocols including preventive measures for PSIs with a high risk of occurring, how to respond to them, and where to report them, and forms that can be filled if they occur. This would not only make it easier for care providers to understand the occurrence of PSIs in their absence but could also help foster a sense of ownership in dealing with PSIs among users and their families. This study has some limitations. First, since the data were collected through self-administered questionnaires, there might be reporting biases such as a social desirability bias to not report unfavorable events to subjects or the occurrence of PSIs unnoticed by the subject because staff from other agencies already responded. Future research must conduct a prospective longitudinal survey on PSI occurrence in homecare by combining self-reports and chart reviews to better understand patient safety in homecare. Finally, this study aimed to survey PSI occurrence in homecare; therefore, the causes of PSIs were not assessed. Further research is required to examine the patient safety outcomes of collecting and sharing PSIs within and between HCN agencies.

Nonetheless, these findings may be valuable for understanding patient safety characteristics in homecare settings. Further research is necessary to explore the causes of PSIs to develop effective strategies for patient safety in homecare settings.

## Figures and Tables

**Table 1:** Characteristics of homecare nursing (HCN) agencies and homecare nurses. (HCN agencies = 27, Homecare nurses = 193).

	Mean (SD) [Range]		
HCN agencies			
Full-time equivalent HCN	6.3	(2.9)	[2.5–12.0]
Visits in one day per HCN	3.8	(2.0)	[0.5–20.0]
PSIs reported over three months	6.3	(5.8)	[0–26.0]
Homecare nurses			
Age	45.5	(8.0)	[26–69]
Sex [female]	179	(92.7)	
Position			
Manager	27	(14.0)	
Chief	15	(7.7)	
Staff	143	(74.1)	
Length of service as homecare nurse	7.88	(6.1)	[1–23]
Average number of visits per day	3.8	(2.0)	[0.5–20]
Number of incident reports reported in the previous year [0–5 cases]	166	(86.0)	
Recognition of the effectiveness of sharing PSI information between HCN agencies for home care patient safety [*effective*]	183	(94.8)	
Experience sharing PSI information between other HCN agencies [*yes*]	55	(28.5)	

**Table 2:** Characteristics of patient safety incidents (PSIs) and clients who experienced PSIs (n = 139).

	Total (n = 139)			Reporting Form of PSI						*p*
				Formal (n = 44)			Informal (n = 95)			
	n (%) or Mean (SD) [Range]									
Types of PSIs										
Fall	61	(43.9)		11	(25.0)		50	(52.6)		0.003
Medication error	35	(25.2)		14	(31.8)		21	(22.1)		0.293
Injury while offering care	7	(5.0)		6	(13.6)		1	(1.1)		0.004
Injury without fall	5	(3.6)		1	(2.3)		4	(4.2)		1
PSIs related to office processing (i.e., Unvisited)	5	(3.6)		4	(9.1)		1	(1.1)		0.035
PSIs related to drip route or urinary catheter	5	(3.6)		3	(6.8)		2	(2.1)		0.326
Aggravation of symptoms by clients’ bad compliance to recuperation	4	(2.9)		1	(2.3)		3	(3.2)		1
Occurrence or deterioration of pressure ulcer	4	(2.9)		0	(0.0)		4	(4.2)		0.307
Accidental consumption	3	(2.2)		2	(4.5)		1	(1.1)		0.235
PSIs related to tube feeding	3	(2.2)		1	(2.3)		2	(2.1)		1
Other	6	(4.3)		3	(6.8)		3	(3.2)		0.432
Context										<0.0001
Occurred during a visit by HCNs	42	(30.2)		25	(56.8)		17	(17.9)		
Occurred while HCNs were not visiting	97	(69.8)		19	(43.2)		78	(82.1)		
Impact of PSIs on clients										0.66
Unplanned admission/consultation was not required	110	(79.1)		36	(81.8)		74	(77.9)		
Unplanned admission/consultation was required	29	(20.9)		8	(18.2)		21	(22.1)		
Contributor of PSIs										<0.0001
Homecare nurse	34	(24.5)		24	(54.5)		10	(10.5)		
Clients/Informal care givers	105	(75.5)		20	(45.5)		85	(89.5)		
Identifier of PSIs										0.143
Homecare nurse	65	(46.8)		25	(56.8)		40	(42.1)		
Clients/Informal care givers	74	(53.2)		19	(43.2)		55	(57.9)		
Clients’ characteristics										
Age	75.8	(22.7)	[0–101]	75.4	(24.6)	[0–101]	75.9	(22.0)	[0–101]	0.901
Sex [Female]	82	(59.0)		30	(68.2)		52	(54.7)		0.193
ADL										
Transfer motion [Independence]	69	(49.6)		14	(31.8)		55	(57.9)		0.006
Excretion motion [Independence]	73	(52.5)		16	(36.4)		57	(60.0)		0.016

Note. PSIs: Patient Safety Incidents; ADL: Activities of Daily Living.

## Data Availability

The data presented in this study are not publicly available because of privacy restrictions.
